# RNA-seq reveals novel CircRNAs involved in breast cancer progression and patient therapy response

**DOI:** 10.1186/s12943-020-01198-2

**Published:** 2020-04-15

**Authors:** Christophe Nicot

**Affiliations:** Department of Pathology and Laboratory Medicine, University of Kansas Medical Center, 3901 Rainbow Boulevard, Kansas City, KS 66160 USA

In two studies recently published in Molecular Cancer, [1, 2] used RNA deep sequencing in control pairs of breast cancer and adjacent noncancerous to identify non-coding circRNAs involved in the pathogenesis of breast cancer. These studies revealed autophagy-associated circCDYL and a novel circRNA circSEPT9 significantly upregulated in breast cancer tissues and involved in breast cancer pathogenesis.

Increased expression of circCDYL in the tumor tissues and serum of breast cancer patients was significantly correlated with higher tumor burden. In fact, CircCDYL was the most abundant circRNA present in the serum from metastatic breast cancer patients compared to early breast cancer and benign patients. Kaplan-Meier analysis indicated that patients with high circCDYL had a poorer disease-free survival and real time kinetics of serum circCDYL in metastatic breast cancer patients during chemotherapy found that reduction of circCDYL is positively correlated to a better sensitivity to clinical therapy, suggesting that high levels of circCDYL is indicative of a poorer clinical response to therapy. Mechanistically circCDYL promotes breast cancer disease progression by targeting microRNA miR-1275. Using TargetScan Human, miRNA.org and miRNAWalk, authors identified seven autophagy-associated genes as potential targets of miR-1275. Among those, only ATG7 and ULK1 expression was decreased as a result of miR-1275 sponge. Authors then confirmed that circCDYL promotes proliferation via autophagy flux in breast cancer cell lines in vitro and in an in vivo orthotopic animal model.

CircSEPT9 is generated by back-splicing exon2 of the SEPT9 gene. Bioinformatic analyses revealed E2F1-binding sites on the SEPT9 promoter and SEPT9 is the target gene of transcription factor E2F1. Furthermore, several binding sites of EIF4A3, a factor involved in pre-mRNA splicing, were detected in the upstream and downstream region of the circSEPT9 mRNA transcript via Circinteractome. These results suggest that both E2F1 and EIF4A3 can modulate expression of circSEPT9 in breast cancer cells. Importantly, expression of circSEPT9 and clinical pathological characteristics. The results displayed that the expression of circSEPT9 was positively correlated with T, N and TNM stages, but not with other clinical pathological parameters. Kaplan-Meier survival curves showed that patients with high expression of circSEPT9, N2–3 stage and TNM stage II/III had a shorter overall survival than those with low expression of circSEPT9, N0–1 stage and TNM stage I. Down-regulation of circSEPT9 induces cell cycle arrest, apoptosis and autophagy in triple negative breast cancer cells (TNBC). Then, using TargetScan and miRanda databases, authors found that circSEPT9 targets miR-637. Because miR-637 directly target LIF, overexpression of circSEPT9 was associated with an increased expression of the LIF-STAT3 pathway. Finally, authors demonstrated that expression of CircSEPT9 accelerates the growth and metastasis of xenograft tumors in vivo. circSEPT9 significantly associated with the clinical stage of TNBC patients. In summary, both circSEPT9 and circCDYL can serve as important prognostic and predictive factors for breast cancer patients disease progression and therapy response. The presence of these circRNAs in liquid biopsy may provide clinicians real-time information for decision-making in personalized treatments.

## Data Availability

Not applicable.
